# Annotated Differentially Expressed Salivary Proteins of Susceptible and Insecticide-Resistant Mosquitoes of *Anopheles stephensi*

**DOI:** 10.1371/journal.pone.0119666

**Published:** 2015-03-05

**Authors:** Sonam Vijay, Ritu Rawal, Kavita Kadian, Kamaraju Raghavendra, Arun Sharma

**Affiliations:** 1 Protein Biochemistry and Structural Biology Laboratory, National Institute of Malaria Research (ICMR), Sector-8, Dwarka, New Delhi, India; 2 Insecticide Resistance Laboratory, National Institute of Malaria Research (ICMR), Sector-8, Dwarka, New Delhi, India; CSIR;, INDIA

## Abstract

Vector control is one of the major global strategies for control of malaria. However, the major obstacle for vector control is the development of multiple resistances to organochlorine, organophosphorus insecticides and pyrethroids that are currently being used in public health for spraying and in bednets. Salivary glands of vectors are the first target organ for human-vector contact during biting and parasite-vector contact prior to parasite development in the mosquito midguts. The salivary glands secrete anti-haemostatic, anti-inflammatory biologically active molecules to facilitate blood feeding from the host and also inadvertently inject malaria parasites into the vertebrate host. The *Anopheles stephensi* mosquito, an urban vector of malaria to both human and rodent species has been identified as a reference laboratory model to study mosquito—parasite interactions. In this study, we adopted a conventional proteomic approach of 2D-electrophoresis coupled with MALDI-TOF mass spectrometry and bioinformatics to identify putative differentially expressed annotated functional salivary proteins between *An*. *stephensi* susceptible and multiresistant strains with same genetic background. Our results show 2D gel profile and MALDI-TOF comparisons that identified 31 differentially expressed putative modulated proteins in deltamethrin/DDT resistant strains of *An*. *stephensi*. Among these 15 proteins were found to be upregulated and 16 proteins were downregulated. Our studies interpret that *An*. *stephensi* (multiresistant) caused an upregulated expression of proteins and enzymes like cytochrome 450, short chain dehyrdogenase reductase, phosphodiesterase etc that may have an impact in insecticide resistance and xenobiotic detoxification. Our study elucidates a proteomic response of salivary glands differentially regulated proteins in response to insecticide resistance development which include structural, redox and regulatory enzymes of several pathways. These identified proteins may play a role in regulating mosquito biting behavior patterns and may have implications in the development of malaria parasites in resistant mosquitoes during parasite transmission.

## Introduction

In developing countries, malaria is one of the serious arthropod borne disease causing mortality and morbidity. *An*. *stephensi* Liston (*Diptera*:*Culicidae)*, a major urban malaria vector spans throughout the Middle East and South East Asia, contributes to 12% of malaria cases annually [1,2]. In spite of the various efforts to control malaria transmission, still this fatal disease is responsible for millions of deaths. Absence of an effective vaccine, emergence of multi drug resistance in *Plasmodium* parasite [3,4] and multiple insecticide resistance in mosquitoes [5,6] accentuates the need for novel effective malaria control strategies.

In spite of the development of resistance, various insecticides and insecticide treated nets (ITNs) are being used as malaria control measures in public health system. Therefore, the threat of insecticide resistance and its implications is now a greater challenge. Genetic and proteomic factors and over use of all major groups of insecticides are responsible for rapid development of resistance among malaria vectors [7]. There are various known proteins/enzymes e.g. esterases, monooxogenases and glutathione S-transferases that known to be involved in the development of resistance against various insecticides in the *Anopheles* vectors [8]. Previous studies have reported effect of DDT on parasite development and showed blood fed insecticide resistant mosquitoes showed low survival rate after exposure of insecticides [9]. Other studies also showed impact of insecticide resistance on expression of salivary gland proteins in resistant acetylcholine allele of *Culex sp* [10]. Therefore, in order to understand the plausible role of expressed functional proteins of insecticide resistance mosquitoes in the development of parasite, physiological changes in the mosquito and various important enzymes of metabolic pathways, further knowledge of various proteins is required.

Salivary glands are an important organ of *Anopheles* mosquito, because of its main role in the transmission of the infective stage of the malaria parasite and in parasite vector interactions. Mosquito salivary proteins are important because they contain various bioactive factors like anti-coagulation factors, platelet aggregation inhibition proteins and immunosuppressive proteins that help the mosquito to overcome homeostasis and blood feeding [10,11]. It is known that insecticide resistance may also impact on the feeding habit of mosquito and vector competence [12]. Therefore, it is important to elucidate the role of functional proteins that are directly annotated in the insecticide resistant species in the development of parasites.

In this study, we adopted a conventional proteomic approach of 2D-electrophoresis coupled with MALDI-TOF mass spectrometry and bioinformatics to identify putative differentially expressed annotated functional salivary proteins between *An*. *stephensi* (susceptible) and *An*. *stephensi* (multi-resistant) strains. Expressed annotated functional salivary proteins or peptides that are upregulated/downregulated in insecticide resistant *An*. *stephensi* mosquitoes may have some role in various parasite development studies of the malaria parasite. These annotated proteins may be helpful in explaining the behavior of resistant mosquitoes toward the development of resistance and may lead to a search for a diagnostic protective antigen for novel malaria control strategies.

## Materials and Methods

### Mosquitoes

The two strains of *An*. *stephensi* namely *An*. *stephensi* susceptible (S) and *An*. *stephensi* multi-resistant (R) used in this study were reared and maintained in our insectary. These strains were maintained at 27°C ± 2°C with 70% ± 10% relative humidity with photoperiods of 12:12 (light/dark) hours. Adult mosquitoes were maintained on a resin and 10% sucrose solution.

### Development of insecticide resistant strain

A multi-insecticide resistant strain of *Anopheles stephensi* was used for proteomics studies. Briefly, the wild mosquitoes were collected from field sites. After collection they were identified and checked for insecticide resistance using WHO prescribed methods (adult susceptibility test). After test, DDT/Deltamethrin/malathion resistance strain of *An*. *stephensi and* susceptible strain *were* maintained separately. This resistant strain was established in the laboratory by selection to deltamethrin at every generation to WHO diagnostic doses and carried out according to WHO procedure [13]. These strains are being tested quarterly against all 3 insecticides and respectively cyclic colonies are being maintained.

To maintain these susceptible and resistant strains properly, insecticide susceptibility test was executed on 3–4 day old adult sugar fed mosquitoes according to WHO recommended doses. This multiple insecticide resistant strain is found resistant to DDT 4% (79%), malathion 5% (54.5%) and deltamethrin 0.05% (21.6%). The resistant ratio (RR) of multiresistant strain of *An*. *stephensi* was against DDT (1.91), Malathion (2.17) and deltamethrin (0.93). *An*. *stephensi* susceptibility strain was taken as a control. Only those strains were selected as susceptible that found to be 100% mortality after exposed to WHO recommended diagnostic doses.

### Preparation of salivary gland extracts (SGE) for 2D gel-electrophoresis

Salivary glands were dissected from 200 adult sugar fed (3–4 days old) female *An*. *stephensi* mosquitoes each (susceptible and resistant) using fine needles. Dissected salivary glands were ultrasonicated in lysis buffer (Tris 50mM, 150mM NaCl, 1% NP40 with protease inhibitors pH 7.4) for 3 pulses of 20 sec each on ice and homogenized sample were centrifuged at 5000 rpm for 10 min at 4°C (Complete, Roche Diagnostics, Germany). Debris were removed and supernatant was stored at -20°C. The protein concentrations of the SGEs were quantified with the Bradford reagent (Sigma Aldrich). For 2D electrophoresis firstly the SGE samples were desalted and cleaned using the ReadyPrep 2D Cleanup Kit (Bio-Rad), as per the manufacturers’ protocol. Thereafter, cleaned SGE samples were resuspended in 2D ReadyPrep rehydration buffer (8M urea, 2M thiourea, 2% CHAPS, 50 mM Dithiotheritol (DTT), 0.2% Bio-Lyte 3/10 ampholyte, and Bromophenol blue (trace).

### 2D electrophoresis

Salivary gland protein samples were immobilized on IPG strips (17 cm) of pH 3–10 (Linear, Bio-Rad) using a Protean IEF Cell (Bio-Rad) and kept for isoelectric focusing with a default cell temperature of 20°C and a maximum current of 50 mA/strip. Briefly, 300 μl sample in rehydration buffer were thawed and each of them were pipetted as a line along the edge of a channel. IPG strips were gently placed (gel side up) on to the sample and finally layered with 2–3 ml of mineral oil. The sample supernatants were rehydrated on IPG strips at 20°C overnight after which IEF was run in three steps: 1. 250V for 20 min Linear; 2. 10000V for 2.5 hrs Linear; 3. 10000V for 5–7 hrs and 40000 V-hrs in Rapid mode. After IEF run the strips were equilibrated in equilibration buffer I (6M urea, 0.375M Tris-HCl, pH 8.8, 2% SDS, 20% glycerol and 2% DTT) and equilibration buffer II (6M urea, 0.375M Tris-HCl, pH 8.8, 2% SDS, 20% glycerol and 2.5% iodoacetamide for 10 min. The second dimension was carried out on 12% SDS-PAGE on Mini Protean cell (Bio-Rad). The 2D gels were silver stained with FOCUS-FAST silver^TM^ stain (G-Biosciences) after the run to visualize the spots. Stained gels of both the susceptible and resistant strains were scanned and analyzed using ImageMaster 2D Platinum 7.0 software (GE Healthcare Life Sciences).

### In-gel extraction and trypsin digestion of proteins for MALDI analysis

Differential annotated protein spots among 2 strains of *An*. *stephensi* (susceptible and resistant) were excised automatically using a computer assisted ProPic work station (Genomic Solutions). These spots were digested automatically using a Pro Prep protein digestion station (Genomic Solutions). Briefly, all spots were destained using destaining solution for 10 minute intervals (3–4 times) until the gel pieces become translucent white. The gels were dehydrated using acetonitrile. Dried gel pieces were rehydrated with 10 mM dithiotreitol DTT in 100 mM ammonium bicarbonate and alkylated with 55mM iodoacetamide. After 45 min incubation with occasional vortexing, the gel pieces were incubated with 50mM ammonium bicarbonate and rehydrated with acetonitrile for 10 mins and dried the gel pieces using speed vac concentrator. Digestion buffer (50mM NH_4_HCO_3_ (pH: 8.5), 5 mM CaCl_2_, and trypsin (proteomic grade, Roche diagnostics) was added to dried gel pieces and incubated overnight at 37°C. Finally, peptides were extracted thrice by one change of 20 mM NH_4_HCO_3_ and three changes of 5% formic acid in 50% acetonitrile (20 min for each change). The dried pepmix was suspended in TA buffer and spotted onto the MALDI plate after mixing it with matrix solution.

### MALDI-TOF MS analysis

Above digested premix samples were first desalted and concentrated on C18 Zip Tips (Millipore, USA). Desalted peptides samples were mixed with α-cyano-4-hydroxycinnamic acid matrix (50% aqueous acetonitrile and 0.2% trifluoroacetic acid) prepared in 1:1 ratio and the resulting 2 μl was spotted onto the MALDI plate. After air drying the samples, it was analyzed on MALDI TOF/TOF UltraFlex III mass spectrometer (Bruker Daltonics, Bremen, Germany). Maldi TOF analysis was carried out in positive-ion reflectron mode of 500–3000m/z detection range using FLEX control software. Further analysis was performed on FlexAnalysis TM software for obtaining the peptide mass fingerprint (Bruker-Daltonics). For peptide selection, a parameter of mass range was set to 900–3000 Da. Further spectra analysis and peak detection was done using MASCOT Wizard Program (Matrix Science UK).

### Database search

MALDI-MS and computational analysis of differentially expressed proteins was carried out using Mascot software (Matrix Science). Briefly, the peaks obtained in the peptide mass fingerprint were analyzed by MASCOT peptide mass fingerprinting search using NCBInr, Swiss prot database for identification of the proteins. Raw data were analyzed in mascot peptide mass fingerprinting (PMF) using parameters: fixed modification (carbamidomethyl), variable modification (Methionine oxidation), trypsin as an enzyme, peptide tolerance: variable from 50 ppm to 500ppm, Missed Cleavages: 1 or 2. All the searches were performed against different *Anopheles* species, *Aedes* and *Culex* mosquito databases (NCBInr/Swiss prot) and also against *An*. *stephensi* databases submitted in VectorBase (https://www.vectorbase.org/organisms/anopheles-stephensi/). Proteins analyzed by PMF were manually validated on basis of scores, % sequence coverage and peptides match. Further, all the significant proteins/peptides were analyzed using various bioinformatics programme includes SMART programme (http://smart.embl-heidelberg.de/) [14], SIGNAL P 4.1, (http://www.cbs.dtu.dk/services/SignalP/) [15], BLAST P and GO analysis (http://www.geneontology.org/) to find out their probable function.

## Results

### Comparative 2D gel electrophoresis of *An*. *stephensi* (susceptible and resistant) SGE

**Fig 1:**
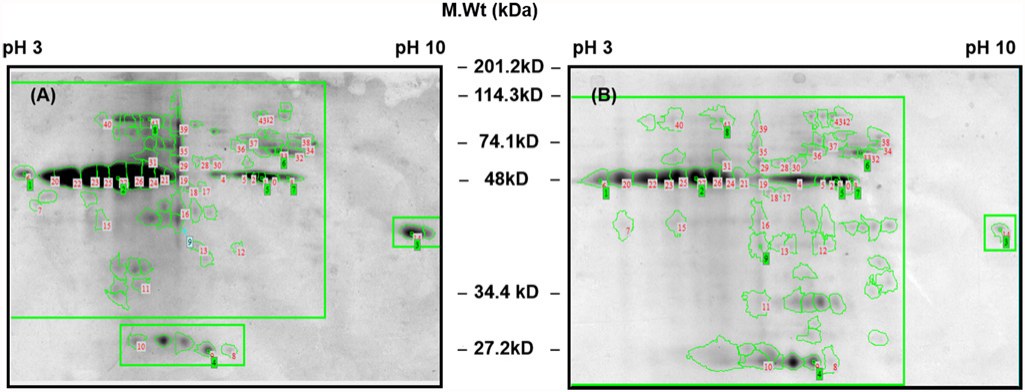
Representative 2D gel picture of *An*. *stephensi* salivary gland proteins. Molecular weight (kDa) is shown in the middle of gel. (A) Silver-stained gel of *An*. *stephensi* susceptible strain numbered from 1 to 43 using ImageMaster 2D Platinum 7.0 software (B) Silver-stained gel of *An*. *stephensi* multi resistant strain numbered from 1 to 43 using ImageMaster 2D Platinum 7.0 software.

**Fig 2:**
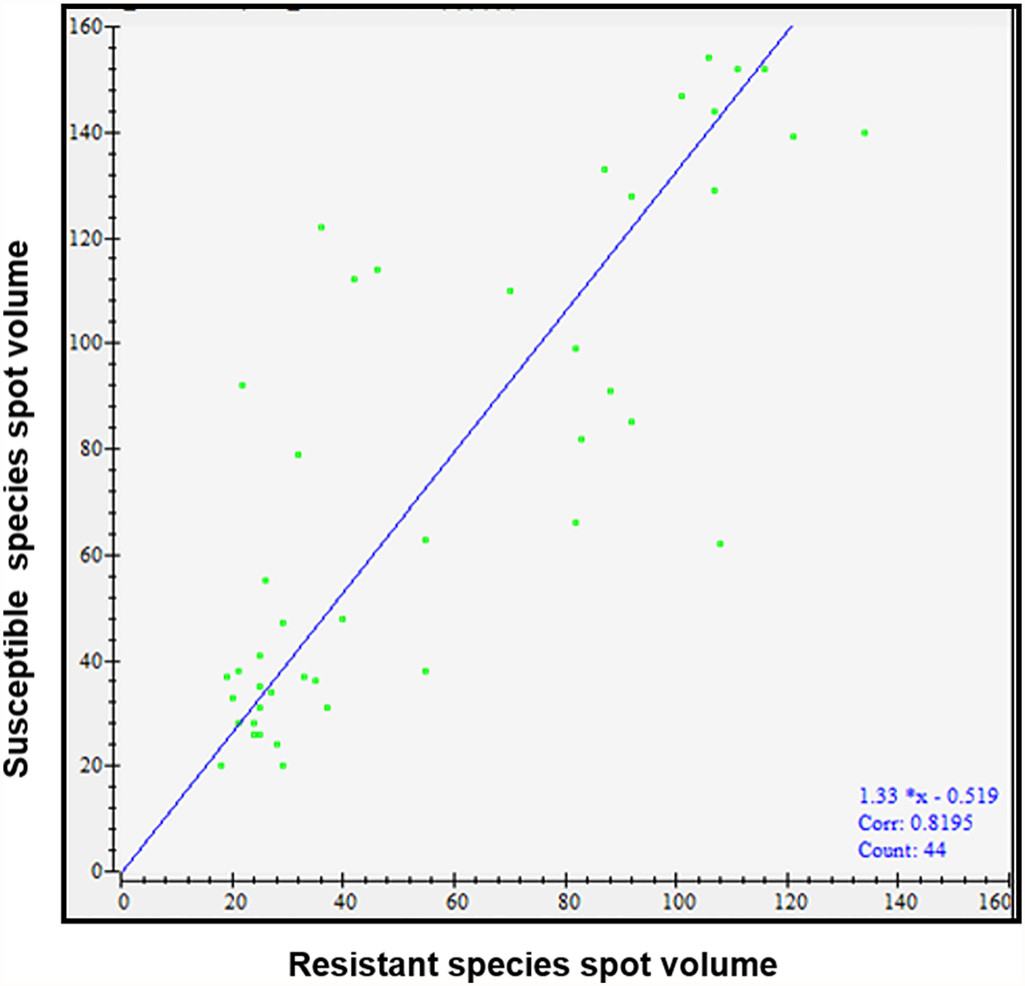
Scatter plot showing volume of all protein spots of *An*. *stephensi* salivary gland. Blue line shows linear regression. X axis represents the volumes of protein spots in *An*. *stephensi* susceptible species and Y axis represents volumes of protein spots in *An*. *stephensi* resistant species. Correlation coefficient was calculated and indicated at the bottom.

The salivary proteins expression profile between susceptible (S) and multi-resistant strain (R) of an urban malaria vector *An*. *stephensi* sugar fed mosquitoes was analyzed using comparative 2D- electrophoresis coupled with MALDI-TOF mass spectrometry. SGE samples of *An*. *stephensi* susceptible (S) and multi-resistant strain (R) were separated on 17cm IPG strips (pH range 3–10) and 12% polyacrylamide in 2D gels. A total 168 spots between a pI range of 3–9 and MW 25–110 kDa were detected by ImageMaster 2D Platinum software. Depiction of the gel picture of total protein identified in *An*. *stephensi* susceptible (S) and multi-resistant strain (R) by 2D-electrophoresis is shown (Fig. 1A and 1B). Among 168 spots, 44 spots were validated in *An*. *stephensi* susceptible species and 36 spots were validated in *An*. *stephensi* resistant species during differential expression analysis. Further among PMF of 44 major spots, 28 spots were found to be similar and 16 annotated spots were found to be different in both susceptible and resistant strain of *An*. *stephensi*. These comparisons of differential spots in susceptible and resistant species were on the basis of pixel volume of each spot. In Fig. 2, volume of spots of both species is shown as a scatter plot which was calculated on the basis of spot area and intensity. Here, it showed the linear distribution between the spot volumes. The correlation coefficient between susceptible and resistant species of *An*. *stephensi* was >0.81 (Fig. 2).

### Identification of *An*. *stephensi* salivary proteins by MALDI-TOF mass spectrometry

**Fig 3:**
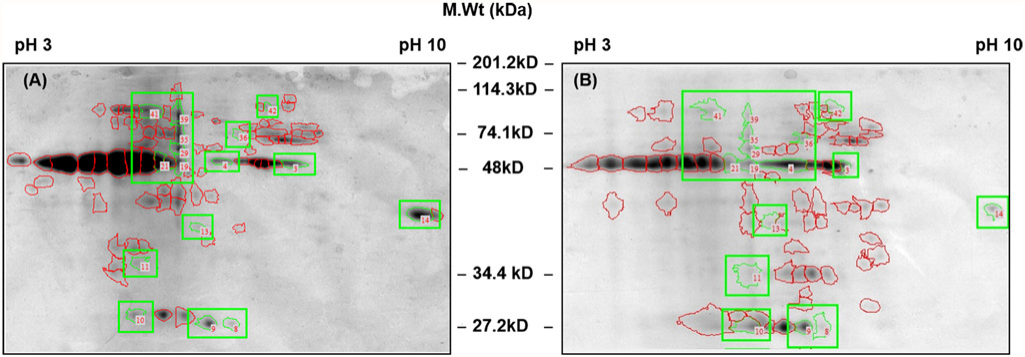
Differential spot selection of salivary gland protein in *An*. *stephensi* species. Representation of the protein spots showing upregulated and downregulated expression in *An*. *stephensi* strains are marked by green line. Molecular weights (kDa) are shown in the middle of 2D gel. (A) Silver-stained gel of *An*. *stephensi* susceptible strain (B) Silver-stained gel of *An*. *stephensi* multi-resistant strain. Depiction of total 16 spots marked using ImageMaster 2D Platinum 7.0 software. Downregulated proteins: Match spot id (3,13,14,21,29,35,39); Upregulated proteins: Match spot id (4,8,9,10,11,19,36,41,42).

In the present study, only those spots which depicted the differential protein expression were explored for comparison between resistant species and susceptible species. Among 16 annotated spots, 9 major spots (spot numbers 4, 8, 9, 10, 11, 19, 36, 41 and 42) were shown to be up regulated in resistant species in comparison to susceptible species (Fig. 3A) and 7 spot (spot numbers 3, 13, 14, 21, 29, 35 and 39) were shown to be down regulated in resistant species (Fig. 3B). These annotated spots were majorly detected of molecular weight range 25–100 kDa and a pI range of 5–9. 2D spots of all upregulated and downregulated spots were further subjected to MALDI-TOF analysis and the peak list (xml) data obtained were analyzed by Mascot algorithm using peptide mass fingerprinting. PMF of all 16 annotated spots led to the identification of 31 different proteins using NCBInr and Swiss Prot database. All the identified proteins correspond to different spots with fold expression values are depicted (Table 1). A total of 15 proteins correspond to 9 differential upregulated spots were identified and their molecular weight, sequence coverage, functions with their respective spots are shown in table (Table 2). A total of 16 proteins correspond to 7 spots were identified as downregulated spots in resistant strain and all the information about each protein i.e. sequence coverage, molecular weight etc with spot numbers are summarized in Table 3.

**Table 1:** Annotated total identified proteins spots with their fold expression values in *An*. *stephensi* multi resistant strain.

Spots number	Fold Expression values	Expression	Protein
4	7.21	Up regulated	(a) Dehydrogenase/reductase SDR family protein
8	2.17	Up regulated	(a) AGAP013415-PA
			(b) Contact-activation-inhibitor protein hamadarin
			(c)Hypothetical protein
9	1.64	Up regulated	(a) GI18065
			(b) Sorting nexin-6
			(c)Tyrosyl DNA phosphodiesterase
10	4.04	Up regulated	(a) molybdenum cofactor synthesis protein 3
			(b) SG1 like 3 salivary protein
11	3.38	Up regulated	(a) Cytochrome P450
			(b) Cyclin-C
			(c)Piwi
			(d) Protein N-terminal glutamine amidohydrolase
19	3.65	Up regulated	No significant protein found
36	1.78	Up regulated	(a) GD24233
41	1.67	Up regulated	No significant protein found
42	3.93	Up regulated	(a) AGAP011997-PA
13	0.24	Downregulated	(a) GL20884
			(b) Conserved hypothetical protein
14	0.25	Downregulated	No significant protein found
21	0.46	Downregulated	(a) Hypothetical protein
			(b) Hypothetical protein
			(c) Hypothetical protein
			(d) AGAP010075-PA
29	0.38	Downregulated	(a) AGAP004469-PB
			(b) Mediator of RNA polymerase II transcription subunit 18
			(c)Ubiquitin carboxyl-terminal hydrolase x4
35	0.38	Downregulated	(a) conserved hypothetical protein
39	0.25	Downregulated	(a) Conserved hypothetical protein
			(b) Yippee putative zinc-binding protein
			(c)26S proteasome non-ATPase regulatory subunit
			(d) ND3 gene product (mitochondrion)
			(e) AGAP005129-PA
			(f) GG20396
3	0.24	Downregulated	No significant protein found

**Table 2:** Summary of upregulated salivary proteins identified in the SGE of *An*. *stephensi* multi resistant strain by MALDI-TOF MS/MS.

S.No.	Accession Number	Protein Name	Peptides	M.wt	∑Coverage	Function	Spot no.
**1.**	GI:195580271	GD24233(Similar to *Drosophila simulans*)	12	18163	65%	Nucleic acid binding	36
**2.**	GI:195118380	GI18065 (Similar to *Drosophila mojavensis*)	10	40758	36%	Transferase activity	9
3.	GI:347971632	AGAP013415-PA (similar to *An*. *gambiae)*	2	10949	29%	Unknown function	8
4.	GI:6224814	Cytochrome P450 (similar to *Culexpipienspallens*)	2	14383	23%	Oxidoreductase activity	11
5.	GI:158294330	Dehydrogenase/reductase SDR family protein(similar to *An*. *gambiae)*	6	34259	19%	Oxidoreductase activity	4
6.	GI:21314941	Contact-activation-inhibitor protein hamadarin(*An*. *stephensi*)	5	19100	19%	Odorant binding protein	8
7.	GI:170037883	Sorting nexin-6 (similar to *Culexquinquefasciatus*)	9	46777	17%	Cell signaling	9
8.	GI:170030570	Molybdenum cofactor synthesis protein 3 (similar to *Culexquinquefasciatu*s)	2	48929	13%	Catalytic activity	10
9.	GI:118792848	AGAP011997-PA(similar to *An*. *gambiae)*	3	35219	10%	ATP Binding	42
10.	GI:157135767	Cyclin-C (similar to *Aedesaegypti*)	2	31664	9%	Cell cycle and transcription	11
11.	GI:170032395	Piwi (similar to *Culexquinquefasciatus*)	4	94829	8%	Protein binding	11
12.	GI:158293025	Protein N-terminal glutamine amidohydrolase (similar to *An*. *gambiae)*	2	25166	7%	Hydrolase activity	11
13.	ASTM004242-PA	Hypothetical protein	6	65402	5%	Unknown function	8
14.	ASTM002501-PA	Tyrosyl DNA phosphodiesterase	9	110393	12%	Hydrolase activity	9
15.	ASTM013042-PA	SG1 like 3 salivary protein	7	45829	18%	No conserved domain	10

**Table 3:** Summary of down regulated salivary proteins identified in the SGE of *An*. *stephensi* multi resistant strain by MALDI-TOF MS.

S.No.	Accession Number	Protein Name	Peptides	M.wt	∑Coverage	Function	Spot No.
1.	GI|195173302	GL20884 (similar to *Drosophila persimilis*)	6	7310	86%	Unknown function	13
2.	GI:170072329	Conserved hypothetical protein (similar to *Culexquinquefasciatus*)	3	6731	69%	Unknown function	39
3.	GI:170056586	Conserved hypothetical protein (similar to *Culexquinquefasciatus*)	7	16918	54%	Unknown function	13
4.	ASTM020854-PA	Yippee putative zinc-binding protein (*An*. *stephensi*)	4	13323	48%	DNA binding protein	39
5.	ASTM013984-PD	Hypothetical protein (*An*. *stephensi*)	4	9682	47%	Unknown function	21
6.	ASTM012002-PA	Hypopthetical protein (*An*. *stephensi*)	4	9230	38%	Unknown function	21
7.	GI:347971968	AGAP004469-PB (similar to *An*. *gambiae)*	5	13618	35%	Binding protein	29
8.	ASTM002073-PA	Hypothetical protein (*An*. *stephensi*)	4	18695	25%	Unknown function	21
9.	GI:157014218	AGAP010075-PA (similar to *An*. *gambiae)*	5	18221	28%	Unknown function	21
10.	GI:55233705	26S proteasome non-ATPase regulatory subunit (*An*. *gambiae*)	6	43409	27%	Protein binding & regulation	39
11.	GI:5834918	ND3 gene product (mitochondrion)(similar to *An*. *gambiae)*	2	13734	23%	Oxidoreductase activity	39
12.	GI:158292620	AGAP005129-PA(similar to *An*. *gambiae)*	4	30588	20%	catalytic activity	39
13.	GI:158563993	Mediator of RNA polymerase II transcription subunit 18 (similar to *An*. *gambiae)*	2	24022	17%	regulation of transcription	29
14.	GI:157110219	Ubiquitin carboxyl-terminal hydrolase x4 (similar to *Aedesaegypti*)	4	54523	17%	Protease activity	29
15.	gi|194883280	GG20396(similar to Drosophila erecta)	8	84290	16%	cell-cell recognition	39
16.	GI:170054535	conserved hypothetical protein (similar to *Culexquinquefasciatus*)	7	74134	13%	protein binding	35

### Differential protein in multi resistant strain of *An*. *stepehensi*

**Fig 4:**
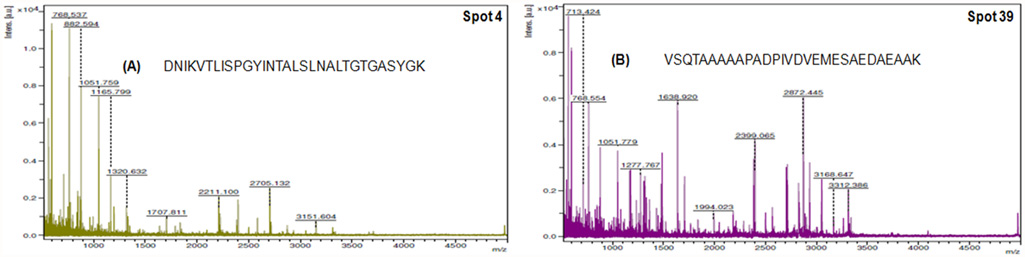
Peak spectrum analyzed by MALDI-TOF in *An*. *stephensi* multi-resistant species. (A) Representative PMF spectrum of over expressed protein i.e Short-chain dehydrogenase reductase (DNIKVTLISPGYINTALSLNALTGTGASYGK) (spot no. 4) (B) Representative PMF spectrum of down regulated protein i.e 26S proteasome non-ATPase regulatory subunit (VSQTAAAAAPADPIVDVEMESAEDAEAAK) (spot no.39).

**Fig 5:**
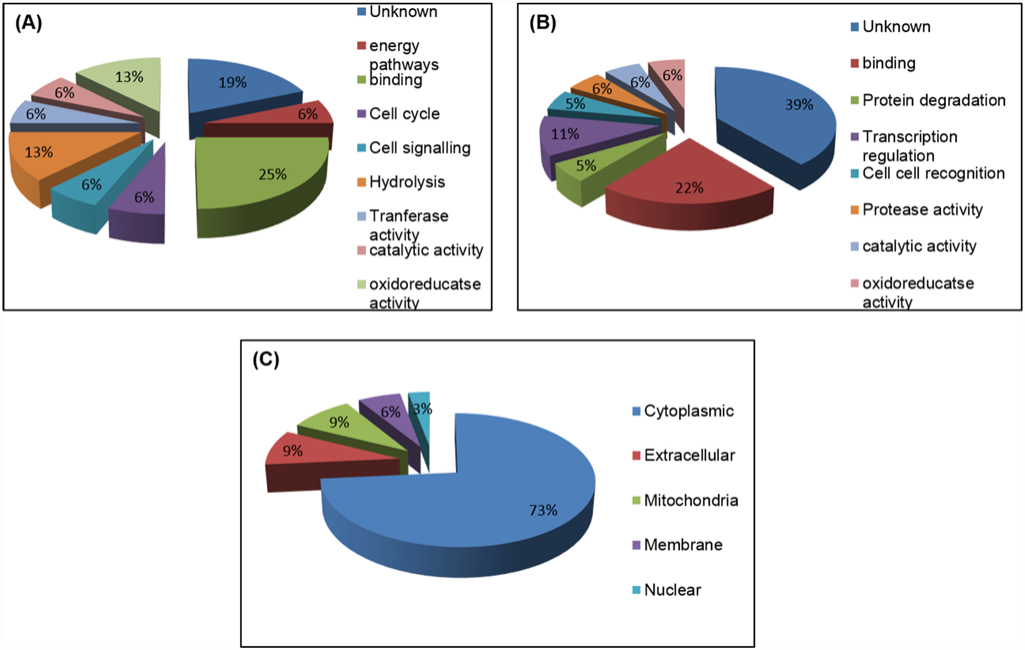
Illustration of identified upregulated & downregulated salivary proteins analyzed by gene ontology tool. (A) GO function of identified upregulated proteins. (B) GO function of identified downregulated proteins. (C) GO component of identified proteins.

Detailed functional analysis of these 31 identified proteins was further explored using various bioinformatics algorithms. The most important upregulated protein identified was short chain dehydrogenase reductase (spot 4) with fold expression 7.21 and cytochrome P450 (spot 11) with fold expression 3.38. Peak spectrum of short chain dehydrogenase is shown with peptide sequence (Fig. 4A). Others upregulated protein identified are hamadarin, Sorting nexin-6, molybdenum cofactor synthesis protein 3, Cyclin-C, Piwi protein, phosphodiesterase, SG1 protein etc. Among the downregulated proteins expression, proteins identified were mostly hypothetical proteins, Yippee putative zinc-binding protein, 26S proteasome non-ATPase regulatory subunit, ND3 gene product (mitochondria), S-methyl-5′-thioadenosine phosphorylase, Mediator of RNA polymerase II transcription subunit 18, Ubiquitin carboxyl-terminal hydrolase x4 etc. Peak spectrum of 26S proteasome non-ATPase regulatory subunit is shown with peptide sequence (Fig. 4B). Mostly upregulated proteins are identified with putative function of oxido-reductase activity, catalytic activity, cell signaling, protein binding etc and mostly down regulated proteins found of unknown function, protein binding, transcription regulation, cell-cell recognition etc. These identified proteins could be depicted into biological function of each upregulated and downregulated protein with percentage as a pie chart was shown (Fig. 5A and 5B) and cellular location of each protein was also depicted (Fig. 5C). Peak spectrum of tryptic peptides of all upregulated spots and downregulated spots are shown respectively (S1 and S2 Figs.).

### Network analysis of upregulated protein

**Fig 6:**
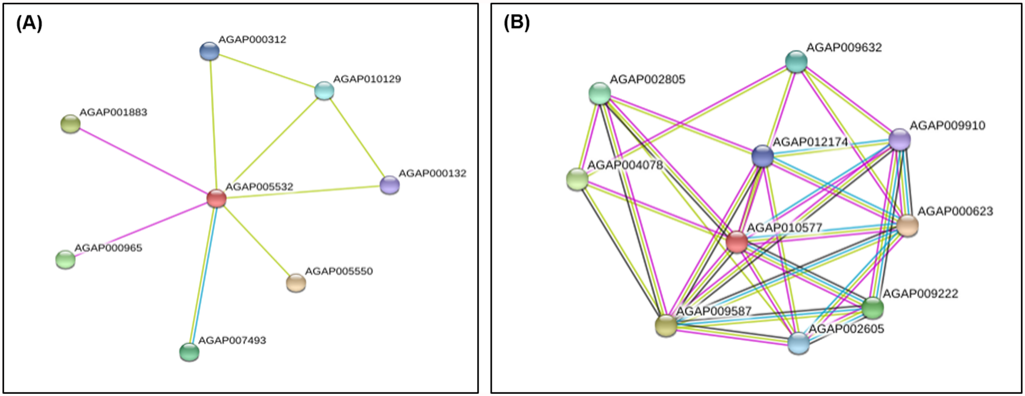
Evidence view of upregulated proteins in resistant strain showing interaction with other proteins. (A) SDR protein (AGAP005532) (B) Tyrosyl DNA phosphodiesterase. Different line colors represent the types of evidence for the association. Green color depicts neighborhood; red color: gene fusion; pink color: experiments; light green color: text mining; blue color: co-occurrence; dark blue color: co-expression; purple color: homology. Circle nodes indicated different proteins.

Protein-protein interaction of most important upregulated protein i.e. short chain dehydrogenase reductase in multi-resistant strain of *An*. *stephensi* was analyzed using STRING 9.1 (http://string-db.org/) [16]. Evidence view of SDR protein was found to interact with other protein of *An*. *gambiae* i.e. AGAP005550, AGAP007493 (oxidoreductase activity), AGAP001883 (nucleotide binding), AGAP000965 (nucleotide binding), AGAP010129, AGAP000312 and AGAP000132 (Fig. 6A). Similarly evident view of another upregulated protein Tyrosyl DNA phosphodiesterase was shown (Fig. 6B). This was shown to interact with AGAP000623 (DNA ligase activity), AGAP009587 (DNA binding), AGAP004078 (DNA binding), AGAP009222 (DNA ligase activity), AGAP002805 (DNA binding), AGAP009632 (ATP binding), AGAP002605 (DNA repair), AGAP012174, AGAP009910 (Helicase activity).

## Discussion

Study of salivary gland proteins of malaria vector are of fundamental importance and a point of attraction because of their role in feeding, malaria parasite transmission and secretion of biologically active molecules [17–19]. Recent *An*. *stephensi* genome analysis [20] and various other studies [21] that have been carried out in *An*. *stephensi* salivary glands represent amino terminal sequences and the proteins in relationship to the transcriptomic analysis [22–23]; however none of these studies were able to assign a specific functional role to most of the gene products. Moreover, no study has directly explained the role of annotated proteins involved in function of parasite development/mechanism in insecticide resistant mosquitoes except the study on *Culex* [10]. In our earlier studies we have carried out *An*. *stephensi* salivary glands proteomics analysis which depicted the plausible role of various directly derived proteins [24]. Therefore, here in this study our main objective was to identify, compare and elucidate a plausible role of the annotated differentially expressed salivary proteins in insecticide resistant vectors using proteomic studies. To achieve this objective we analyzed role of putative functional annotated salivary gland proteins of susceptible *An*. *stephensi* mosquitoes and compared it with multi-resistant *An*. *stephensi* mosquitoes using 2D-electrophoresis, MALDI-TOF mass spectrometry and bioinformatics approaches for a plausible functional role in development of malaria parasites.

We have compared the expression profile of salivary gland proteins between susceptible and insecticide resistant strains of *An*. *stephensi* which have a same genetic background. Differential protein profiles of the SGEs of *An*. *stephensi* (susceptible) and of *An*. *stephensi* (Resistant) were examined based on the fold abundance values and the annotated spots. Our results showed identification of total 168 spots and among them 9 spots were identified as upregulated and 7 spots were identified as downregulated in multiresistant species of *An*. *stephensi*. This led to the identification of 31 proteins: 15 upregulated proteins and 16 downregulated proteins from the SGE of *An*. *stephensi* (resistant) which explain the alteration of expressed salivary gland protein by DDT/Deltamethrin resistance and that may play a role in parasite growth and development.

The identification of an upregulated protein cytochrome P450 (spot 11) similar to *Culex* mosquito was identified with 23% sequence similarity. It is known that cytochrome P450 allows insects to metabolize insecticides at a higher rate [25,26]. It is a kind of metabolic resistance which usually through the overproduction of detoxification enzymes. Earlier also its role was identified and validated by transcriptomics studies however in this study functional annotation of cytochrome P450 role was identified by proteomic studies directly [27,28]. This suggests that this upregulated expression of cytochrome P450 in resistant strain of *An*. *stephensi* may play a major role in insecticide resistance [8]. Another upregulated protein (spot 4) was identified as short chain dehydrogenase-reductase (SDR) that was found to be similar to *An*. *gambiae* with 19% sequence coverage. It has been reported that various roles of short chain dehydrogenase in lipid, amino acid, carbohydrate, hormone, and xenobiotic metabolism as well as in redox sensor mechanisms [29,30]. Surprisingly fold expression of this protein (7.21) was found the highest among all over expressed proteins. From these studies our result suggests that this upregulated protein in resistant strain may be associated with xenobiotic detoxification. Evidence view of SDR protein also shows interactions with the other associated proteins and such protein—protein interactions may further important for signaling studies. Next protein, the factor named hamadarin (spot 8) (fold expression: 2.17) that inhibits activation of the plasma contact system was also found to be upregulated in resistant strain [31]. Isawa proposed the function of this protein in biting of mosquitoes for efficiently sucking of the blood meal and this may be by attenuating the host’s acute inflammatory responses [31]. This protein was also demonstrated to be the part of D7 family, an odorant binding protein [31].

One cytoplasmic protein with 10% sequence similarity (AGAP011997) (spot 42) was found to be over expressed with 3.90 fold expression value. In this one conserved domain i.e. MR domain (multiple resistant domain) along with ATPases was found. Cyclin C (spot 11), was also found to be upregulated whose function is to control of cell cycle and regulation of gene transcription. Piwi protein (spot 11) that found to be upregulated in resistant species, its function is to control transposon and also helps to target genes through gene-drive systems based on transposons [32]. One hypothetical protein (spot 8) was found with RCC1 domain that have important role in regulation of gene expression. This protein was also identified with secreted function and its signal peptide was present at position 1 to 17. This over expressed protein may imply regulation of those genes that involved in resistance mechanism. Tyrosyl DNA Phosphodiesterase protein (spot 9) was next identified protein that was involved in DNA repair mechanism [33]. This upregulated protein with DNA repair function may have a role in some insecticide resistance mechanism. This may involve in repairing function of any alter gene in resistant species caused by any mutation. SG1 protein identified (spot 10) is found to be have secreted function with 4.0 fold expression. Signal peptide of this protein starts at position 1 and ends at position 20. Other upregulated proteins identified have various functions like protein degradation, nucleotide binding etc. This may imply that higher expression of these enzymes in multiresistant insecticide strain may suggest the higher metabolic activity of salivary glands cells and such over expressed proteins might have some role in regulation and in resistance mechanism.

Among downregulated proteins found in resistant species, many of them were of unknown function. Approx 7 proteins of unknown function corresponding to spot no. 13, 21, and 39 were identified. Next in list were various binding proteins that also shown to be down regulated in resistant species. One protein (spot 39) namely Yippee putative zinc-binding protein, its downregulated expression (0.39 fold expression) in resistant species reflected its lower binding capacity. This protein is found to be conserved among all eukaryotes [34]. One protein that role in regulation of transcription of nearly all RNA polymerase II-dependent genes is MED 18 found with 17% sequence similarity was also downregulated in resistant species.

Although this study showed various proteins that were found to be modulated by effect of DDT/Malathion/Deltamethrin insecticides but some important salivary proteins were not detectable in the *An*. *stephensi* strains like D7 protein, apyrase, enolase, transporters protein etc. However, this may be due to our failure to detect small transcripts for short forms of proteins using MALDI-TOF approach or also non detection of all the spots by 2D electrophoresis. Though in our earlier studies, these proteins were identified in *An*. *stephensi* salivary gland using in gel digestion approach [24].

These results emphasize the need of further experimentations to assess the effect of infected blood feeding on salivary gland protein expression profile in susceptible and resistant mosquitoes. We will also find such observations that may explain changes or delayed in oocyst development in resistant mosquitoes to uncover the mechanism of malaria control as a novel tool. Further studies on the role of these annotated proteins in involvement in probing time and biting behavior is required to assess the fitness of resistant mosquitoes for parasite development. Studies on the biting behavior patterns of susceptible and insecticide-resistant mosquitoes, its impact on parasite development and transmission are being carried out in our laboratory for novel control strategies.

## Conclusion

In summary, our initial study constitutes the first study of MS based proteomic approach to identify and compare the protein profiles of *An*. *stephensi* salivary glands of susceptible and insecticide/ pyrethroid multiresistant strains. Through the use of 2D electrophoresis coupled with MALDI TOF, differential expression profile of SGEs annotated protein are examined and assessed by sequence coverage, peptide sequences and by fold expression. Our results depict the differentially regulated proteins in response to insecticide resistance in *An*. *stephensi* multiresistant strain and identified these proteins with major function like xenobiotic detoxification, insecticide resistance, DNA repair, regulation of gene transcription, control transposon etc. Mapping of putative upregulated/downregulated proteins in resistant strains of *An*. *stephensi* may have impact on the biting behavior of mosquitoes to adapt to environment and hence the transmission of malaria parasites. These studies may prove useful information in identification and annotation of genes to better understand the molecular mechanism for a diagnostic tool behind the resistance in *An*. *stephensi* multiresistant strain and furthermore contribute to the novel strategies to control the malaria pathogen transmission.

## Supporting Information

S1 Fig Peak spectrums of upregulated proteins analyzed by MALDI-TOF. Representative PMF spectrum of over expressed proteins of spot no. 8, 9, 10, 11, 19, 36, 41 and 42.
(TIF)

S2 Fig Peak spectrums of downregulated proteins analyzed by MALDI-TOF. Representative PMF spectrum of under expressed proteins of spot no. 3, 13, 14, 21, 29 and 35.
(TIF)
