# Supplementary figures and images for: Annotated Differentially Expressed Salivary Proteins of Susceptible and Insecticide-Resistant Mosquitoes of Anopheles stephensi
Source: PLoS One. 2015 Mar 5;10(3):e0119666. doi: 10.1371/journal.pone.0119666 (PMC4351086; doi:10.1371/journal.pone.0119666)

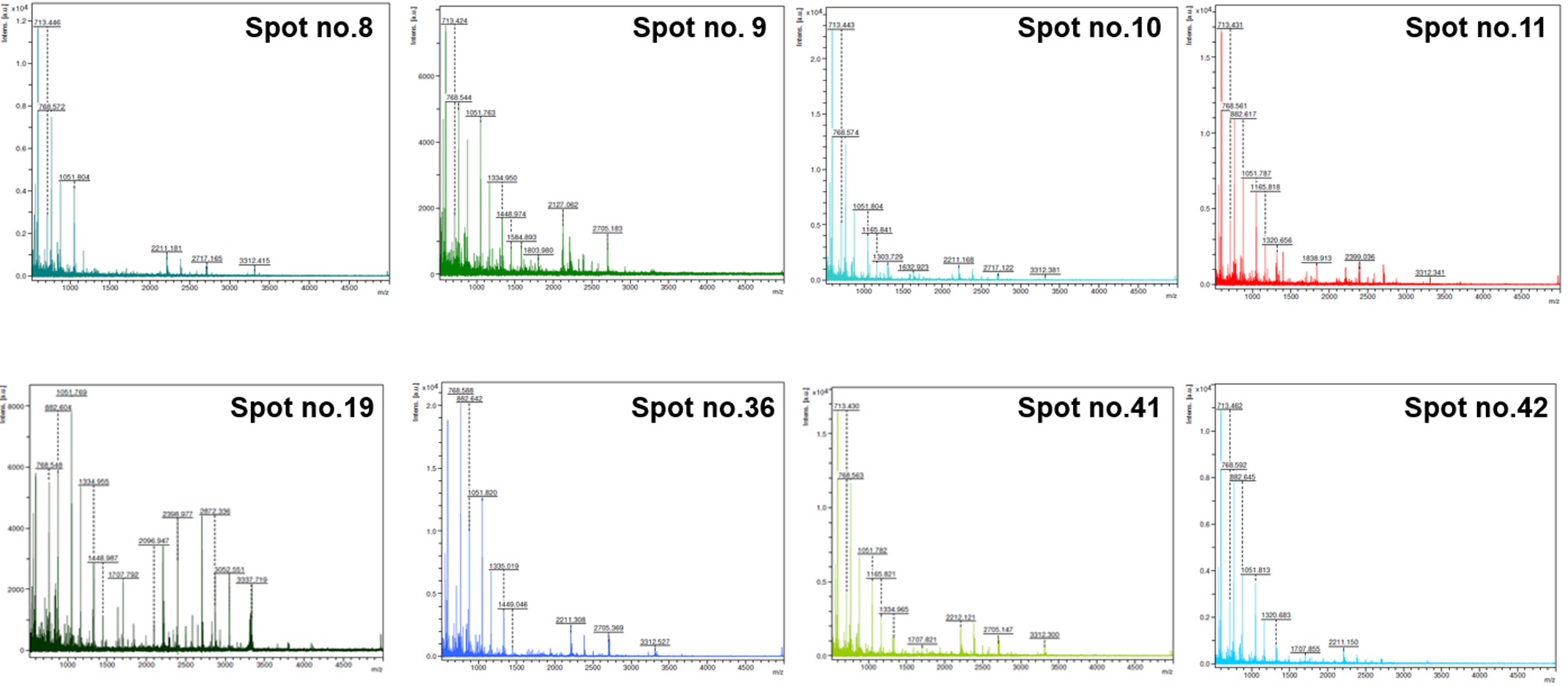

Supplement: S1 Fig — Representative PMF spectrum of over expressed proteins of spot no. 8, 9, 10, 11, 19, 36, 41 and 42. (TIF) [file pone.0119666.s001.tif]

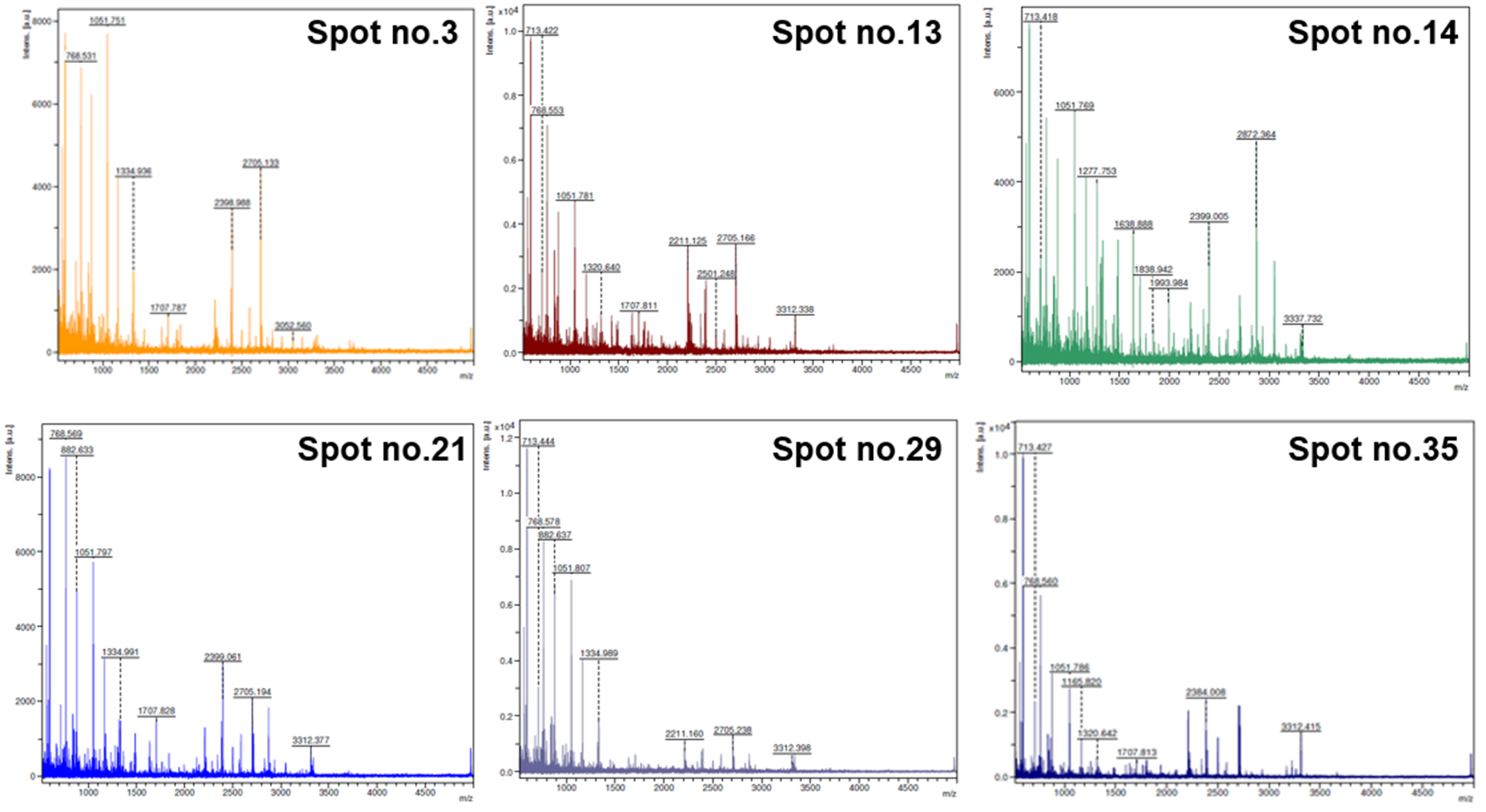

Supplement: S2 Fig — Representative PMF spectrum of under expressed proteins of spot no. 3, 13, 14, 21, 29 and 35. (TIF) [file pone.0119666.s002.tif]
